# Apoptosis like symptoms associated with abortive infection of *Mycobacterium smegmatis* by mycobacteriophage D29

**DOI:** 10.1371/journal.pone.0259480

**Published:** 2022-05-17

**Authors:** Fatema Calcuttawala, Rahul Shaw, Arpita Sarbajna, Moumita Dutta, Saptarshi Sinha, Sujoy K. Das Gupta

**Affiliations:** 1 Department of Microbiology, Sister Nivedita University, Kolkata, India; 2 Department of Microbiology, Bose Institute, Kolkata, India; 3 Division of Electron Microscopy, National Institute of Cholera and Enteric Diseases, Kolkata, India; 4 Department of Physics, Bose Institute, Kolkata, India; University of Montana, UNITED STATES

## Abstract

Mycobacteriophages are phages that infect mycobacteria resulting in their killing. Although lysis is the primary mechanism by which mycobacteriophages cause cell death, others such as abortive infection may also be involved. We took recourse to perform immunofluorescence and electron microscopic studies using mycobacteriophage D29 infected *Mycobacterium smegmatis* cells to investigate this issue. We could observe the intricate details of the infection process using these techniques such as adsorption, the phage tail penetrating the thick mycolic acid layer, formation of membrane pores, membrane blebbing, and phage release. We observed a significant increase in DNA fragmentation and membrane depolarization using cell-biological techniques symptomatic of programmed cell death (PCD). As Toxin-Antitoxin (TA) systems mediate bacterial PCD, we measured their expression profiles with and without phage infection. Of the three TAs examined, MazEF, VapBC, and phd/doc, we found that in the case of VapBC, a significant decrease in the antitoxin (VapB): toxin (VapC) ratio was observed following phage infection, implying that high VapC may have a role to play in the induction of mycobacterial apoptotic cell death following phage infection. This study indicates that D29 infection causes mycobacteria to undergo morphological and molecular changes that are hallmarks of apoptotic cell death.

## Introduction

Bacteriophages, literally meaning ‘bacteria devourers’, are the most abundant and diverse biological entities in the world [1]. There are ~10^31^ phage particles in the biosphere [2]. There is a dictum that phages are ubiquitously found where bacteria thrive, thereby playing a fundamental role in regulating bacterial ecology. They are obligate intracellular bacterial parasites with either a lytic or a lysogenic life cycle [3]. Even though they were discovered in 1915 by Frederick Twort, the nature of the existence of the so-called “*contagium vivum fluidum*”—whether it was liquid or particulate, remained a topic of contention until they were visualized for the first time in the year 1939 with an electron microscope (EM) [4,5]. Several significant milestones were achieved by phage imaging, including their morphological classification [6].

The therapeutic potential of these transmissible bacteriolytic entities was first identified by their co-discoverer, Félix d’Hérelle [7]. Since then, phage research has become the cradle of fundamental and translational biosciences. Since phages can kill their hosts efficiently, microbiologists are becoming increasingly interested in exploiting them as antibacterial agents replacing conventional drugs [1,8]. Phage D29 is one such bacteriophage, which infects diverse mycobacteria such as *Mycobacterium smegmatis* and *Mycobacterium tuberculosis* [9]. Belonging to the family Siphoviridae, it typically exhibits a long non-contractile tail [10]. The resurgence of tuberculosis (TB) and the emergence of excessive drug-resistant (XDR) and pandrug (PDR) resistant strains has spurred renewed interest in the therapeutic use of mycobacteriophages [11]. They can even serve as cornerstones for developing novel diagnostic and preventive strategies [12]. D29 is the prototypical model for a mycobacteriophage as it efficiently adsorbs to the host and begins DNA replication within a few minutes after infection. One-step growth experiments have demonstrated that the length of the latent period, which is the time taken from infection to the onset of lysis, is 30–35 minutes in *M*. *smegmatis* but is 2–3 hours in *M*. *tuberculosis* [13,14]. The rationale for using *M*. *smegmatis* as the host is that it is non-pathogenic and grows substantially faster than *M*. *tuberculosis* [15]. The kinetics of the phage infection cycle is directly co-related to its host’s generation time and is extended in slow-growing bacteria. For instance, DNA synthesis is observed 2–4 minutes after infection in *M*. *smegmatis* but is delayed to 20 minutes in the slow grower, *M*. *tuberculosis* [16]. Recent observations show that *M*. *smegmatis* mc^2^155 is both restriction- and CRISPR-free suggesting that these are positive attributes for discovering phages [17].

Preliminary studies in our laboratory have indicated that phage D29 infection results in ‘host inactivation’ [18]. However, how this happens remains obscure. Recent investigations have revealed that multiple mechanisms could be involved, such as generation of superoxide radicals and induction of thymine less death [18,19]. We undertook the present study to gain an insight into the changes in the host cell upon phage infection. Even though an age-old technique, electron microscopy was resorted to because it is still considered the gold standard for viral ultrastructure studies [20].

Several hurdles confront the utilization of phages for the curtailment of mycobacteria. Rather than recruiting phages directly for treatment, they can be used as platforms for drug discovery [8]. Phages have evolved multiple strategies for interfering with bacterial growth. Understanding the targets that phages use in inhibiting bacterial growth has a clear therapeutic implication. In this study, we focus on the interaction between mycobacteriophage and mycobacteria. Our objective was to develop cytological and molecular tools to understand the changes in bacterial cells once mycobacteriophages attack them.

## Materials and methods

### Bacteria, bacteriophage and media

Infection experiments were performed using *Mycobacterium smegmatis* mc^2^155 (Msm) as the host strain and mycobacteriophage D29, which was obtained as a kind gift from Dr. Ruth McNerney (LSHTM Keppel Street, London, United Kingdom). Middlebrook 7H9 medium (Difco) supplemented with 0.2% glycerol, 0.25% bovine serum albumin (BSA) (HiMedia Laboratories, India), and 0.01% Tween 80 was used for growing mycobacterial cells. Phage infection was carried out in the same medium except that Tween 80 was omitted and 2 mM CaCl_2_ was added to the medium. MB7H9 hard agar plates were used for colony counting. For plaque assay, the hard agar was overlaid with soft agar containing 2 mM CaCl_2_.

### Phage infection of Msm

Phage was amplified by the confluent lysis method followed by suspension in SM buffer (50 mM Tris-HCl, pH 7.4, 100 mM NaCl and 8 mM MgSO_4_). Phage purification was done by performing CsCl density gradient centrifugation, followed by dialysis using a dialysis buffer (50mM Tris-Cl (pH 8.0), 10 mM NaCl, 10 mM MgCl_2_). Msm cells were infected with phage D29 at a multiplicity of infection (MOI) of 1. Aliquots were collected at different time points and centrifuged at 15,700 x *g* for 5 min, the pellet and supernatant fractions were separated and the number of PFUs present in the pellet (infectious centre) and the supernatant (free phage) were determined separately. The sum of the two values at time zero, immediately after phage addition, was considered as the input PFU. The MOI was determined by dividing the input PFU count by the total viable cell count, CFU, which was determined by plating the host cells on the same day.

### Cloning and expression of mycobacteriophage D29 gene *17*

The gene encoding the D29 major head subunit gp17 was PCR amplified using the primers D2917F and D2917R (S1 Table) from mycobacteriophage D29 genomic DNA and subsequently cloned into the BamHI-HindIII site of the expression vector pET-28a (Novagen). The recombinant protein produced possessed a His_6_ tag at its N-terminal end.

### Purification of recombinant protein

The plasmid construct made for over-expressing the protein gp17, the major capsid protein of mycobacteriophage D29, was transformed into *E*. *coli* BL21(DE3). The transformants resistant to kanamycin (50μg/ml) were cultured in the presence of the antibiotic at 37°C. At an OD_600_ of 0.5, isopropyl-β-D-thiogalactopyranoside (IPTG) was added at a final concentration of 0.5 mM. The bacterial cells were induced at 37°C for 3 h. Bacterial pellets were obtained by centrifugation at 10,000 x *g* for 15 min. Cells harvested by centrifugation were lysed by sonication. Protein purification was performed using Ni^+2^-NTA agarose affinity chromatography, under denaturing conditions in the presence of 8M urea, according to standard protocol (Qiagen).

### Raising antibody in rabbit

Polyclonal rabbit antibodies were raised against affinity-purified gp17 protein, which was isolated under denaturing conditions in the presence of 8 M urea. The protein sample was separated on a 10% SDS polyacrylamide gel (SDS-PAGE). The band corresponding to the protein was excised, crushed, and injected into the rabbit intramuscularly. Pre-immune serum was collected before immunization. Two booster doses were administered at 10-day intervals, following which the rabbit was bled. The blood was coagulated, and the serum separated by centrifugation. The specificity of the serum thus obtained was verified by Western blotting as done in an earlier study [21] (S1 Fig).

### Immunofluorescence microscopy studies

At both early and late stages of infection, cells were harvested, washed with PBS, and then fixed in 4% (w/v) paraformaldehyde in PBS for 20 min at room temperature on the cover slip of a microscopic slide. After several PBS washes, blocking was performed using the blocking buffer (3% BSA in PBS) for 15 min. Cells were subsequently treated with primary antibodies in blocking buffer against gp17 at 4°C for 1 h. After several washes with PBS, the cells were incubated with ALEXA -labelled goat anti-rabbit secondary antibodies (Thermo Fisher Scientific, USA) in the blocking buffer at 4°C for 1 h. After several washes with PBS, cells were stained with 4′, 6′-diamidino-2-phenylindole (DAPI) to visualize the nucleic acid. Stained cells were examined by confocal microscopy (Leica TCS SP8).

### Transmission Electron Microscopy (TEM) studies

The interaction between the mycobacteriophage D29 and its host strain was examined by transmission electron microscopy. Infection was performed at an MOI of 1. Samples collected at different stages of infection were negatively stained with 2% uranyl acetate and examined under an FEI Tecnai 12 Biotwin transmission electron microscope (FEI, Netherlands) at an accelerating voltage of 100kV.

### Scanning Electron Microscopy (SEM) studies

For scanning electron microscopy, samples were collected and fixed with glutaraldehyde (1.5% w/v). After centrifugation at 3,300 x *g*, the pellet was dissolved in 20% ethanol solution and spread on the glass slide for drying, mounted on aluminium stubs, coated with gold (Edwards) and photographed with a SEM (FEI Quanta 200).

### Membrane depolarization assay by flow cytometry

Msm cultures were grown overnight at 37°C to an OD_600_ between 0.2–0.3. Infection was done at an MOI of 1 or as mentioned. After treatment, the sample was collected, washed once, and resuspended in 1 ml 1X PBS. Staining of cells was performed using 5 μl of bis-1,3-dibutyl barbituric acid trimethine oxonol (DiBAC_4_, Invitrogen, 0.025 mg/ml in DMSO) [22] followed by incubation for 15 minutes at room temperature in the dark. The intensity of DiBAC_4_ fluorescence was measured using the FACS Verse (BD) system with a 488-nm argon laser for excitation and a 530±15-nm emission filter.

### DNA fragmentation assay by flow cytometry and confocal microscopy

DNA fragmentation was quantified by the TUNEL assay using the ApoDirect kit (BD Biosciences). The enzyme terminal deoxynucleotidyl transferase (TdT) adds fluorescein isothiocyanate deoxyuridine triphosphate (FITC-dUTP) to each 3′-hydroxyl end of fragmented DNA, making it possible to determine the extent of DNA fragmentation by measuring the intensity of fluorescence. For our studies, cells were grown and treated with phage. After washing with PBS, cells were fixed by resuspending them in 1 ml of 1% paraformaldehyde and incubating on ice for 60 min. The cells were then washed with PBS, resuspended in 70% ethanol, and stored at -20°C overnight. The next day, the cells were centrifuged, ethanol was discarded, and the cell pellet was resuspended in 1 ml wash buffer provided in the kit. After two washes using this buffer, the pellet was resuspended in 50 μl of a staining solution comprising reaction buffer, FITC-dUTP, and TdT dissolved in distilled water. The reaction mixture was incubated at 37°C for 60 min, gently mixing the sample after every 15 min. The reaction was arrested by adding 1 ml of rinse buffer from the kit. The rinse was performed twice, followed by the addition of 500 μl propidium iodide/RNase solution and incubation in the dark for 30 min. at room temperature. The PI/RNase treatment reduces RNA-related background and reports the DNA content of the labelled cells. The intensity of fluorescence was measured using the FACSVerse (BD) system. An aliquot of the stained cells was centrifuged, resuspended in 1X PBS, and examined by confocal microscopy (Leica TCS SP8).

### RNA extraction, RT-PCR and qRT-PCR

RNA was extracted from 20 ml of broth culture. Cells were harvested by centrifugation (13,000 x *g*, 5min) and re-suspended in 2 ml TE buffer (10 mM Tris-HCl pH 8, 1 mM EDTA) containing lysozyme at a concentration of 5 mg/ml, followed by incubation at 37°C for 30 min. This step was followed by adding 5 ml TRIzol reagent (Invitrogen), 1ml chloroform, and centrifugation at 13,000 x *g* for 15 min. The aqueous phase was collected and precipitated by adding 0.7 volumes of cold isopropanol, followed by 70% ethanol wash. The pellet was dried and re-suspended in 50 μl of DEPC treated water. Contaminating DNA was removed by DNaseI digestion (Qiagen) and RNA using RNeasy Mini Kit (Qiagen). RNA quality was assessed by agarose gel electrophoresis and A_260/280_ ratio determination.

First-strand cDNA synthesis was done from 400 ng RNA using RevertAid Reverse Transcriptase (Fermentas) by random priming according to the manufacturer’s instructions. One microliter of cDNA, 0.25mM dNTPs, 0.2 pmol/ μl of each primer, and 0.3U of Taq polymerase were used for 25 μl of RT-PCR reaction mixtures. Thermal cycler conditions were: 94°C for 5 min, 30 cycles of 94°C for 30 s, 55°C for 30 s followed by 72°C for 7 min., and a final hold at 4°C. 16S rRNA transcript levels were used as endogenous control. The PCR products were analysed by agarose gel electrophoresis.

Real-time analysis was conducted using Power SYBER^R^ Green PCR Master Mix (Applied Biosystems) in a 7500 Fast Real-Time PCR system (Applied Biosystems). The cycling conditions were: 50°C for 2 min, 95°C for 30 s, 55°C for 30 s, 72°C for 45 s. All the primers used for RT-PCR and qRT-PCR are enlisted in S1 Table.

## Results

### Immunofluorescence microscopic studies of phage-host interaction

The interaction of mycobacteriophage D29 with its host bacterium Msm was visually inspected by means of immunofluorescence microscopy. To generate antisera against the phage we cloned the gene for gp17, the major capsid protein of phage D29 in the *E*. *coli* expression vector pET28a. Following induction of gene expression, the protein was purified under denaturing conditions (S1A Fig). The band corresponding to the purified protein was excised and used to raise antisera in rabbit. The pre-immune and immune sera were then collected from the rabbits and used to perform Western blot analysis using the purified gp17 protein. The Western blot analysis revealed that only the immune sera cross reacted (S1A Fig) but not the pre-immune sera. This result confirmed that the antisera contained anti-gp17 antibodies at the desired level.

**Fig 1:**
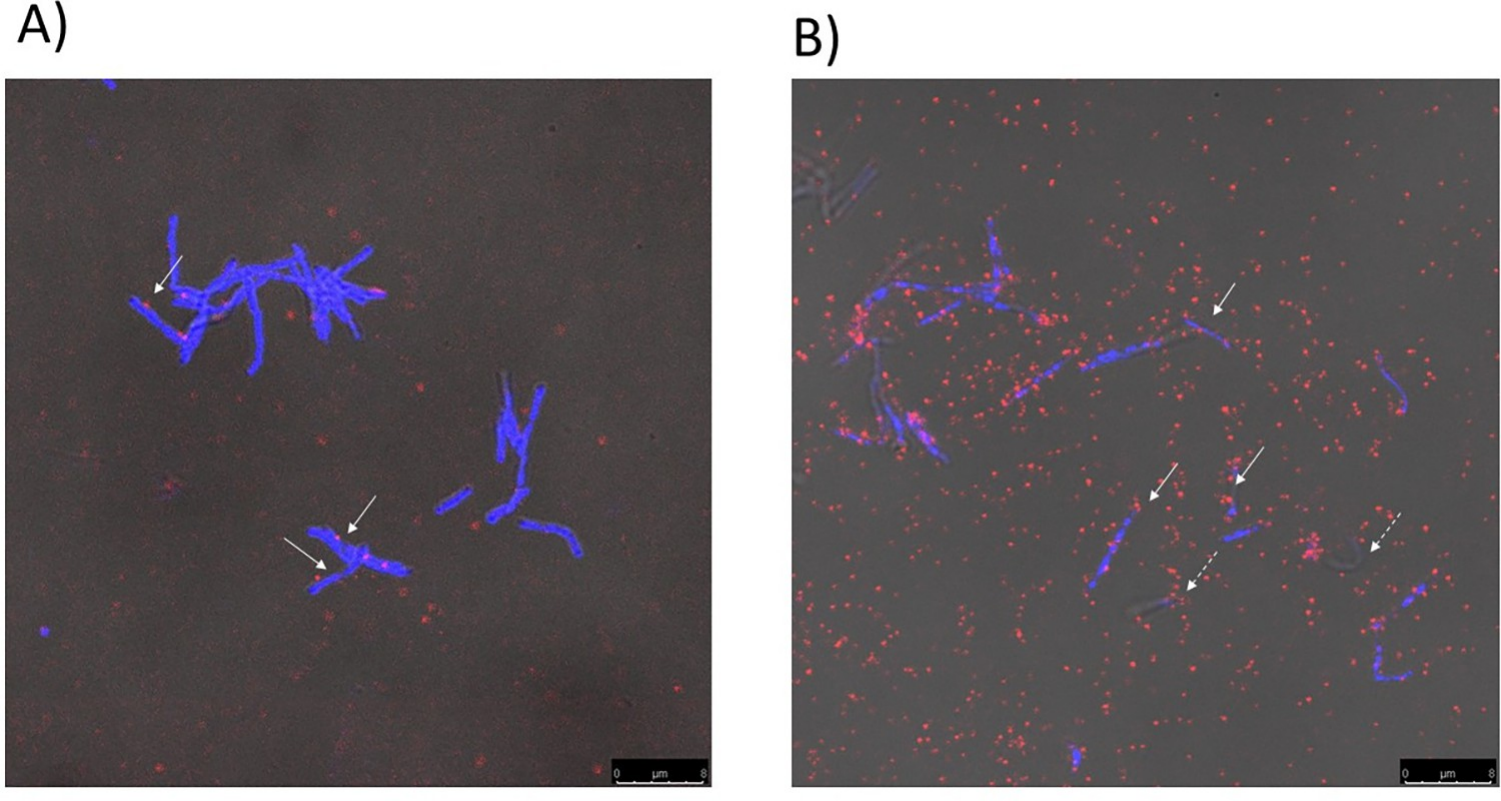
Immunofluorescence microscopy of phage infected Msm cells, A) 30 and B) 120 mins post infection. Phages were identified using a polyclonal rabbit serum against gp17 capsid protein, and anti-rabbit ALEXA conjugated secondary antibody (red). The cells were localized using the nucleic acid staining dye DAPI, (blue). The phase contrast, DAPI and ALEXA-stained images were merged. The scale below represents 7.5 μm. The arrows indicate cells with adsorbed phages. Broken arrows indicate cells that were not stained with DAPI.

We used this antiserum to study phage-bacteria interactions. The bacteria were stained using DAPI (blue) and the phage using antisera-anti-rabbit antibody that was counter stained using ALEXA conjugated anti rabbit secondary antibody. The DAPI and ALEXA-stained images were overlapped with the phase contrast images. The images were acquired at two stages of infection, 30 min and 120 min. At the 30 min time point adsorption is expected to have reached a maximal level as reported in our earlier investigations [18]. At 120 mins. the lytic phase is expected to be at its peak. The results indicate that at the 30 min time point there were relatively few phage particles in the field as compared to 120 min (Fig 1A). About 10% of the bacteria, appeared to have adsorbed the phage (Fig 1A, white arrows). In the 120-minute field we observed a large number of phage particles (Fig 1B). Many of these phage particles were found to be adsorbed on the surface of the host cells. Some of the bacterial particles lacked DAPI stain (broken arrows, Fig 1B) indicating that their nucleoids may have degraded.

### Phage induced morphological changes in mycobacteria by electron microscopy

**Fig 2:**
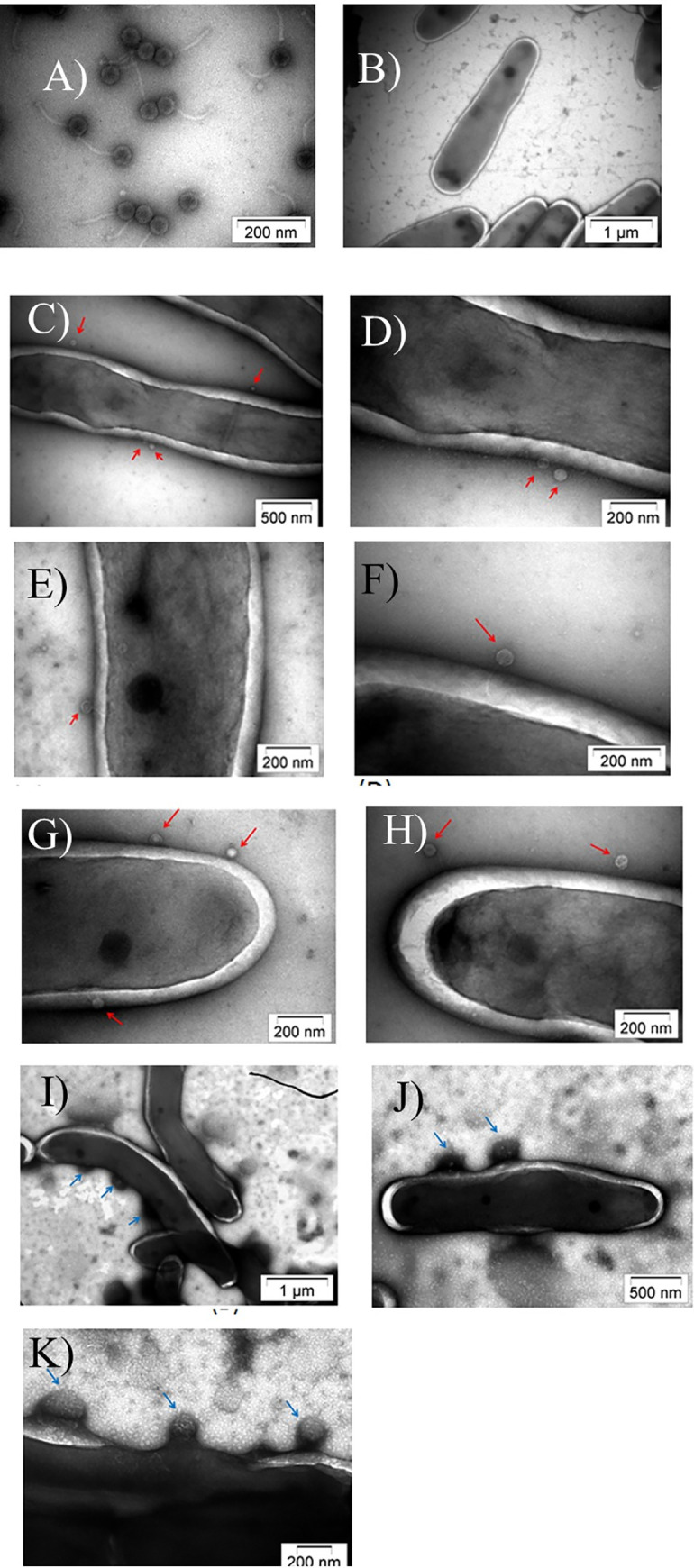
TEM images of the purified phage (A) and the control bacteria (B) taken either separately or after infection (C-K). The adsorbed phage is indicated by red arrows and membrane blebs by blue arrows. (D) is a zoomed image of (C).

**Fig 3:**
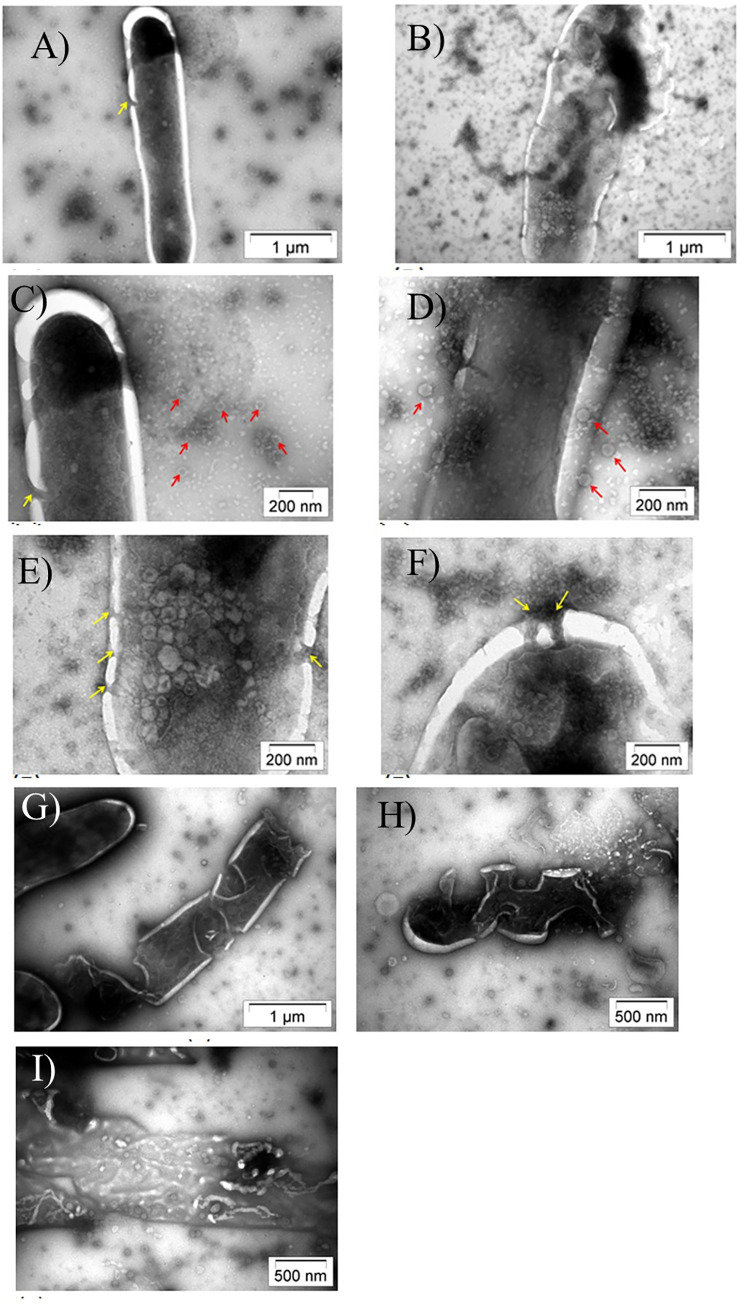
TEM images showing cellular changes during early (A-F) onset and late (G-I) stages of lysis. Red arrows denote phages particles and yellow arrows pore formation in the membrane.

Morphological examination of the phage D29 performed using TEM revealed that it had an icosahedral head with a diameter of ~50 nm and a ~100 nm long tail, at the distal end of which tail fibers were occasionally observed (Fig 2A). The mycobacterial cells measured ~3–4 μm lengthwise and exhibited a typical thick cell envelope (Fig 2B). During the early stages, i.e., 30 mins. post-infection, the adsorption of the phage particles to the host cell was observed. Even though the infection was performed at an MOI of 1, multiple phages were seen adsorbed to the bacterial cell surface (Fig 2C–2H, red arrows). Phages were attached both at the extremities as well as a localized portion on the surface of the mycobacterial cell. Following phage adsorption, penetration of the tail through the thick mycolic acid layer was observed (Fig 2E and 2F). Once the tail pierced the cell wall, the phage retained its normal appearance for a short time. Post-DNA injection, “ghost” phage particles, which are empty capsids, were occasionally found attached to the surface. Distinct protrusions of the membrane, analogous to membrane blebs, were also observed (Fig 2I–2K). At a later stage, i.e., 2 h post-infection, several discrete populations of cells were observed. Some cells exhibited several pores in the membrane (Fig 3A–3F). Leakage of intracellular contents and phage release was observed from these pores. Several mature phages were seen clustered and localized close to the host cell (Fig 3E and 3F). These cells are probably beginning to lyse. Another population consisting mainly of fragmented cells and cellular debris was seen, representing later stages of lysis (Fig 3G–3I).

**Fig 4:**
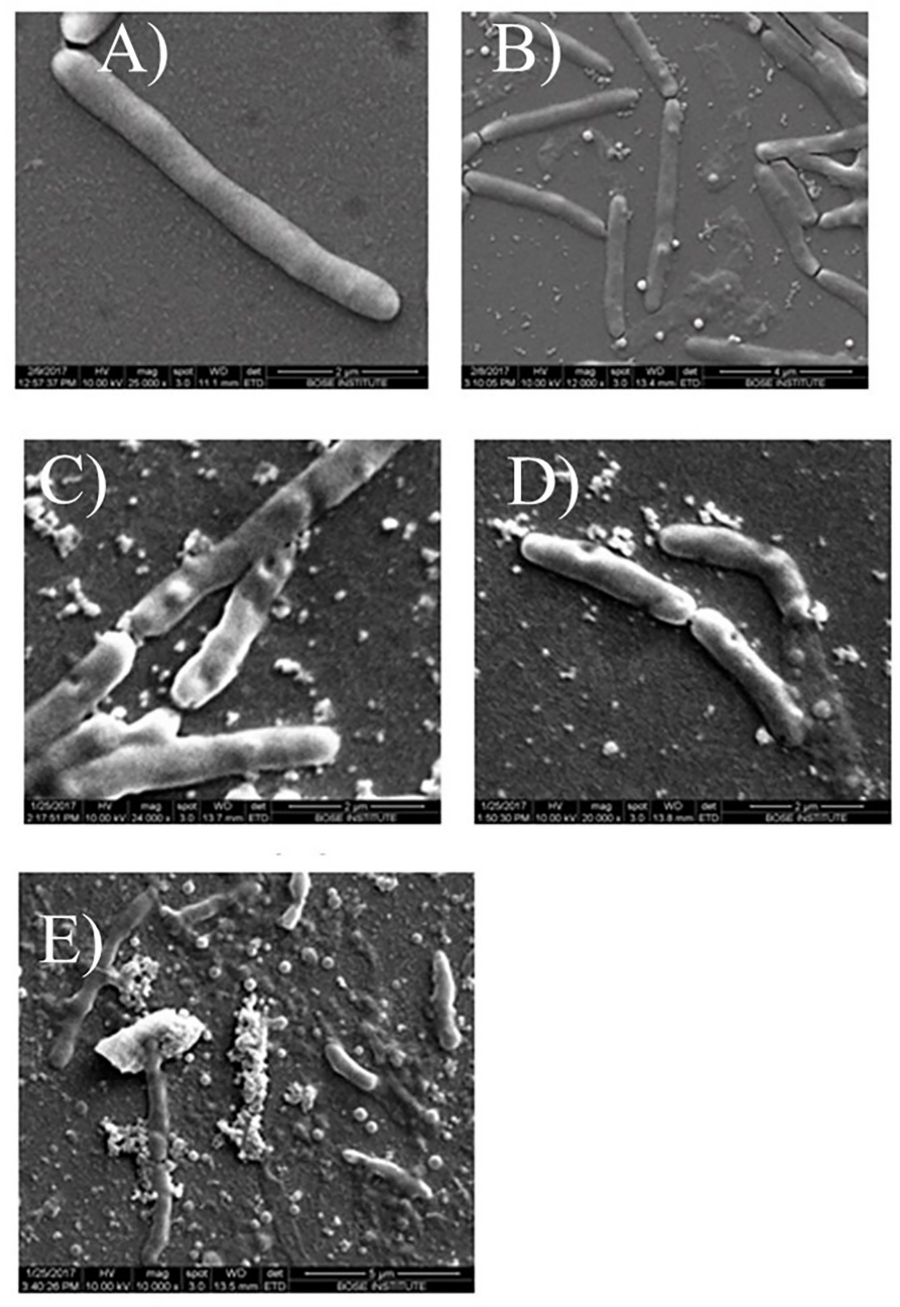
SEM micrographs depicting (A) Msm (B) Phage adsorption (C) Membrane blebbing (D) Pore formation in the membrane and (E) cell lysis.

SEM data correlated well with that of TEM (Fig 4A–4E). In the SEM images too we could see what appears to be adsorbed phage (Fig 4B) and swellings (Fig 4C) that are probably membrane blebs. At the later stage we could see indications of phage release through membrane pores (Fig 4D and 4E).

### Phage infection induces membrane depolarization

**Fig 5:**
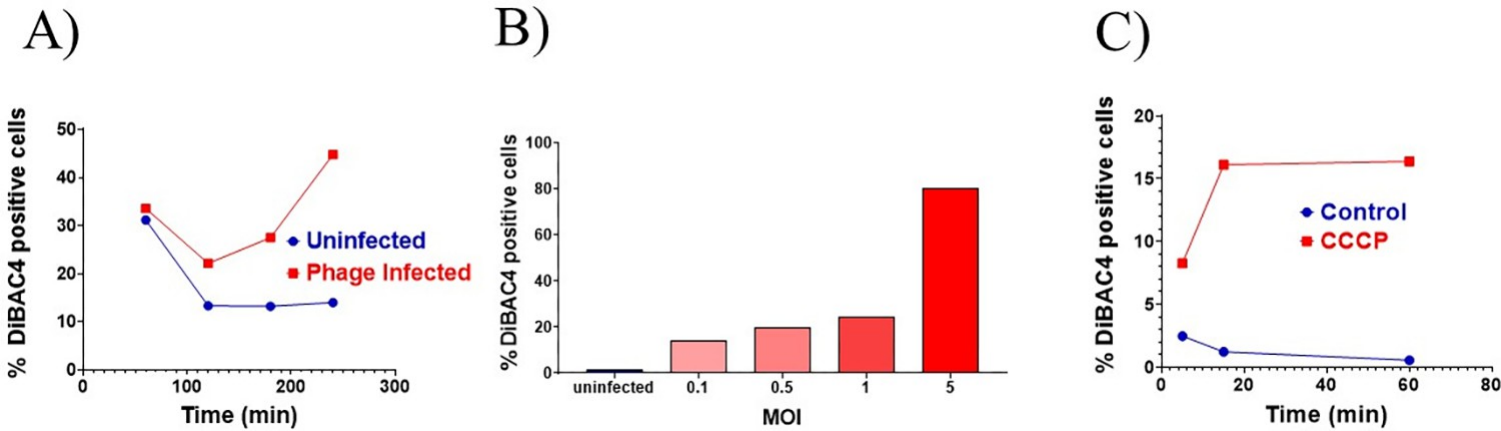
Membrane depolarization assay performed using DiBAC_4_. Fluorescence intensity was measured by flow cytometry using the FITC channel. The experiments were performed using D29 (A, B) as the depolarizing agent or CCCP (C). CCCP was used at a concentration of 3.2 μg/ml.

Membrane depolarization is routinely monitored using the membrane potential sensitive dye DiBAC_4_, which diffuses across depolarized yet intact cell membranes, and binds to lipid-rich intracellular components. We used DiBAC_4_ staining together with flow cytometry to detect fluorescence in individual dying cells (Figs 5 and S2). The result of this experiment reveals a significant increase in DiBAC_4_ fluorescence both as a function of time as well as MOI (Fig 5A and 5B). The time-dependent investigation revealed that there is a decrease in polarization initially. However, after 100 mins. which is the latent period of growth; the depolarization starts to increase at about 180 min. (3 h) and reaches the maximum value at 240 min. (4 h.) (Fig 5A). Thus depolarization begins approximately at the early-middle phase of lytic growth [18]. We also observed an MOI-dependent increase in depolarization (Fig 5B). A control experiment was also performed using the well-known uncoupler and depolarizer, carbonyl cyanide m-chlorophenyl hydrazone (CCCP) [23] (Fig 5C). The results indicate that under the conditions used here substantial time-dependent increase in DiBAC_4_ fluorescence was observed when CCCP was used, which is consistent with previous reports [23,24].

### Phage infection induces TUNEL-detectable DNA fragmentation

**Fig 6:**
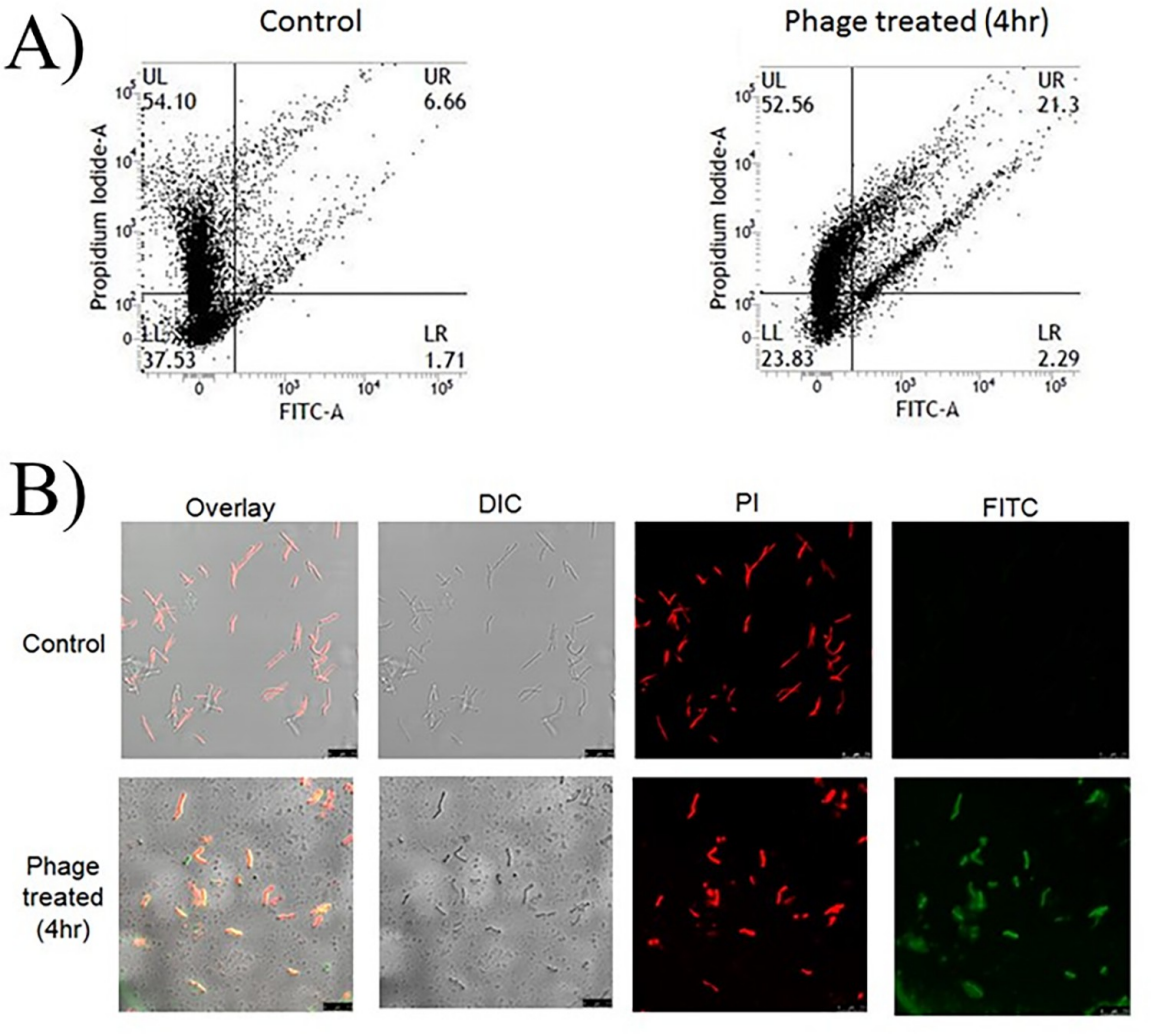
DNA fragmentation detected by the TUNEL assay. (A) FACS based assay and (B) depicts confocal microscopic analysis of phage infected cells. In (A) the quadrants are indicated as UL (Upper Left), UR (Upper Right), LL (Lower Left) and LR (Lower Right). The numbers denote the percentage of cells in the quadrants. The threshold was set using untreated and unstained Msm cells. FITC-fluorescein isothiocyanate, SSC-side scatter. Microscopic analysis of phage-infected and control cells stained with the dyes propidium iodide (PI) and FITC. Scale bars 7.5 μm.

DNA fragmentation was assessed by performing TUNEL assay using the APO-Direct kit. The fluorescence of individual cells was measured by flow cytometry. The positive control was mycobacterial cells treated with hydroxyurea (20 mM) for 8 hours. In the case of phage-treated cells, no appreciable DNA fragmentation was observed at the early stages of infection. However, an increase by about 3 fold after 4 h. in the percentage of TUNEL positive cells (FITC staining cells, UR) (Fig 6A) was observed indicating, enhanced DNA damage. Flow cytometry data was further supported by direct microscopic assessment using PI and FITC to stain the Msm cells. Phage-treated cells stained positively for PI and FITC as compared to control cells which were stained with PI only (Fig 6B).

### Expression profiling of toxin-antitoxin systems of the host upon phage infection

**Fig 7:**
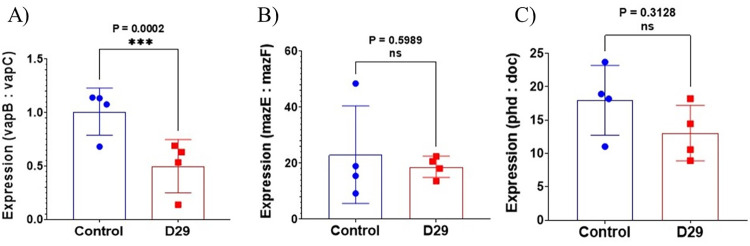
Analysis of TA system gene expression with or without (control) D29 infection for 2h by qRT-PCR. The systems examined are A), VapBC, B), MazEF, C) phd/doc. The expression level of each gene was determined by the comparative C_T_ method after normalizing with a 16S rRNA control. The results of the qRT_PCR experiments are expressed as ratios between the anti-toxin and toxin components. The results are presented as the average of four biological repeat experiments ± S.D. The significance levels (p values) are mentioned and marked with asterix. Non-significant results are marked (ns). The agarose gel electrophoresis analysis of the products (one set) are shown in S3 Fig.

*Mycobacterium smegmatis* contains three putative toxin-antitoxin (TA) systems MazEF, VapBC, and phd/Doc [25]. TA modules comprise a pair of genes usually co-transcribed as an operon, in which the downstream gene encodes the stable toxin, and the upstream one encodes the labile antitoxin [25]. In our study, the expression profile of all the TA systems was analyzed two hours after infection. The results (Fig 7) of the experiments revealed that out of the three systems tested, the VapB-VapC system was significantly affected. The expression ratio (VapB:VapC) was altered considerably in favour of VapC, the toxin (Fig 7A).

## Discussion

The present investigation was undertaken to determine the morphological and physiological changes that mycobacterial cells undergo upon phage infection. In the past, and for a long time, the research with mycobacteriophages possessed a merely phenomenological character, where it was possible to observe productive infection by the phages only by scoring for the efficiency of plaque formation [26]. However, with the advent of fluorescence microscopy, the ‘fluorophages’ provided a visual alternative for mechanistically dissecting the crucial steps of phage adsorption and lysis, leading to plaque formation [27,28]. In this work, we examined phage-host interaction at the single-phage, single-cell level, thereby presenting new tools for studying different properties of infection propagation at the microscopic level. Even though our work is in its infancy, it provides a stepping stone for performing time-lapse experiments under the microscope. This kind of research would give a more precise understanding of infection dynamics and render complete knowledge regarding the propagation of infection in real-time.

Electron microscopy took our studies on understanding phage-host interaction to a fundamentally new level [29]. Right from the past, the mycobacteria-mycobacteriophage system has been considered to be ideal for EM studies because the obstacles confronted using *E*. *coli* and their phages could be overcome by using this model system [29]. The obstacles comprised opacity of the *E*. *coli* cell to electrons, which hindered the observation of the cell interior and the extreme rapidity of lysis, making it impossible to distinguish between different phases of the infection process [30]. However, the electron transparency of mycobacterial cells upon phage attack and the prolonged infection cycle of mycobacteriophages acted as positive attributes for EM studies [31]. We have been able to visualize the complete infection process from the beginning till the end, including phage adsorption, penetration of the non-contractile tail through the thick mycolic acid layer, the morphological changes in the infected cells, including pore formation during the onset of lysis, followed by bursting of the host cells and release of mature phages and intracellular contents. Non-contractile tails are a defining feature of phages belonging to the Siphoviridae family, of which phage D29 is a member [32]. Another striking observation was that the dimensions of the pores in the membrane were intriguingly smaller than the phages emerging from them. Also, the membrane of the infected mycobacterial cells showed structures resembling membrane blebs. Membrane blebs, which are formed when bacteria are exposed to stress [33] can also be found in cells undergoing PCD [34].

PCD is a genetically-determined process characterized by a stereotypical set of morphological hallmarks [35,36]. Our results demonstrated phage infection-induced characteristic changes in the host cells, such as DNA fragmentation, membrane depolarization, and membrane blebbing, that are strikingly reminiscent of eukaryotic apoptosis. Depolarization of the mitochondrial inner membrane is one of the critical events that characterize the intrinsic pathway of apoptosis in eukaryotes [37]. The phage D29 holin-like proteins and the eukaryotic BCL-2 family of proteins seem to utilize similar strategies to disrupt the integrity of the host cell membrane [38]. Bacteria are ancestral to mitochondria, as evidenced by the endosymbiotic theory [39,40]. Thus, considering these similarities, it is tempting to speculate the possibility of convergent evolution of the machinery that controls PCD in prokaryotes and eukaryotes.

Previously, experiments conducted in our laboratory had demonstrated that the decrease in the number of viable cells far exceeded the number of cells that undergo lysis upon phage infection. This phenomenon of death without lysis (DWL) was proposed to involve superoxide radical generation, but other secondary factors remained unexplored [18]. Our results shed light on the possibility of the involvement of PCD in causing DWL. However, the existence of PCD in bacteria seems counterintuitive; the main concern is the benefit of maintaining genes that function to mediate the self-destruction of a unicellular organism. PCD may have an altruistic role under conditions of stress. The primary response of a cell under stressful situations is the induction of DNA repair mechanisms. However, if the damage is insurmountable, with the cost of repair exceeding the cost of building a new cell, a point of no return is reached, and PCD is the last resort option adopted by the cell. The demise of some cells could promote the survival of their siblings [41]. We hypothesize that majority of the cells undergo lysis upon phage infection, but a sub-population undergoes PCD. The small population of surviving cells depends on the nutrients released by the dead cells and may eventually become a nucleus for a renewed population. To the best of our knowledge, this is the first study shedding light on the occurrence of PCD in mycobacteria upon phage infection.

The features associated with abortive infection of Msm by phage D29 suggest that the cells die by PCD. However, we cannot rule out the possibility that the phenomenon we observed is not typically a case of programmed cell death. Whatever the case, this study reveals novel features associated with abortive infection of Msm cells by D29, some of which carry the hallmarks of PCD.

To unravel the genetic mechanism underlying phage-induced mycobacterial PCD, we investigated the involvement of one of its key regulators, the Toxin-Antitoxin (TA) modules. We specifically focused on TA loci because of their ubiquitous presence in bacterial genomes and their increasingly observed roles under stressful conditions [42]. Msm has three TA systems: VapBC, MazEF, and phd/doc. We compared the RNA levels of the toxins and antitoxin genes in Msm uninfected and infected cells. We found a significant effect in the case of VapBC. The VapB RNA content was reduced relative to VapC. In other words, there was a relative increase in the level of VapC. This increase could potentially lead to increased VapC activity in the cell. Increased expression of VapC can have multiple toxic effects on mycobacterial cells. Thus, VapC can have a bacteriostatic effect, as demonstrated in earlier studies [25,43]. VapC overexpression also results in significant changes in the metabolism in Msm cells [44]. In Mtb, VapC cleaves RNAs linked to translation [45]. In Msm, it degrades mRNAs encoding functions related to carbon metabolism [44]. The phage-induced apoptosis-like effects observed here could thus be mediated at least partly due to the dysregulated activity of VapC.

Collectively our results provide a detailed mechanistic insight into the phage-induced mycobacterial cell death. The appearance of different hallmarks of a PCD pathway in phage- infected cells opens an interesting avenue for future research. The potential of this pathway can be tapped by targeting it for future development of a new class of antimicrobials for the treatment of TB.

## Ethical clearances

Polyclonal antibodies were raised in rabbits in the Central Animal Facility of Bose Institute (a CPCSEA, Ministry of Environment, Forest & Climate Change, Government of India guidelines, compliant laboratory) after taking necessary permissions.

## Supporting information

S1 Fig SDS gel electrophoresis (A) and western blot analysis using either anti-gp17 rabbit immune sera (B) or pre-immune sera (C). In (A) the lanes are as follows U (uninduced for gp17 expression), I (induced), P (pellet fraction following centrifugation of lysate), S (soluble supernatant), F (Flow through), E (eluted) and M (mol wt marker). In (B) and (C) the lanes corresponding to the purified protein are marked P. The dilutions performed are indicated above. Arrows point to the band corresponding to gp17 (34 kDa).
(PDF)

S2 Fig Raw data for FACS analysis results. The samples analyzed are D29 infected samples along with controls (uninfected), (A and B). In (A) the changes were followed in a time dependent manner at 1 hr. intervals for 4 hrs. In (B) the same experiment was done in a MOI dependent manner. The time was interval was 2 hrs in each case. In (C) a control experiment was done using the well-known depolarizer DCCP. The exposure times are shown in minutes. Control seta are indicated as C and experimental as (E). The percent cells present in the red zones was used for the interpretation of the results (Fig 5).
(PDF)

S3 Fig Agarose gel electrophoresis of RT-PCR amplicons corresponding to the indicated toxin-anti-toxin systems (Fig 7) M represents DNA size marker. + and–represent amplicons obtained with (+) or without reverse transcription (-).
(PDF)

S1 Table List of primers used. (PDF)

S1 Raw image Unedited gel images corresponding to S1A Fig of supporting information. (TIF)

S2 Raw image Unedited Western blot images corresponding to S2B and S2C Fig of supporting information. (TIF)

S3 Raw image Unedited gel image corresponding to S3A Fig of supporting information. (TIF)

S4 Raw image Unedited gel image corresponding to S4B Fig of supporting information. (TIF)
