# Supplementary material for: Apoptosis like symptoms associated with abortive infection of Mycobacterium smegmatis by mycobacteriophage D29
Source: PLoS One. 2022 May 17;17(5):e0259480. doi: 10.1371/journal.pone.0259480 (PMC9113562; doi:10.1371/journal.pone.0259480)
Supplement: S1 Fig — SDS gel electrophoresis (A) and western blot analysis using either anti-gp17 rabbit immune sera (B) or pre-immune sera (C). In (A) the lanes are as follows U (uninduced for gp17 expression), I (induced), P (pellet fraction following centrifugation of lysate), S (soluble supernatant), F (Flow through), E (eluted) and M (mol wt marker). In (B) and (C) the lanes corresponding to the purified protein are marked P. The dilutions performed are indicated above. Arrows point to the band corresponding to gp17 (34 kDa). (PDF) [file pone.0259480.s001.pdf]

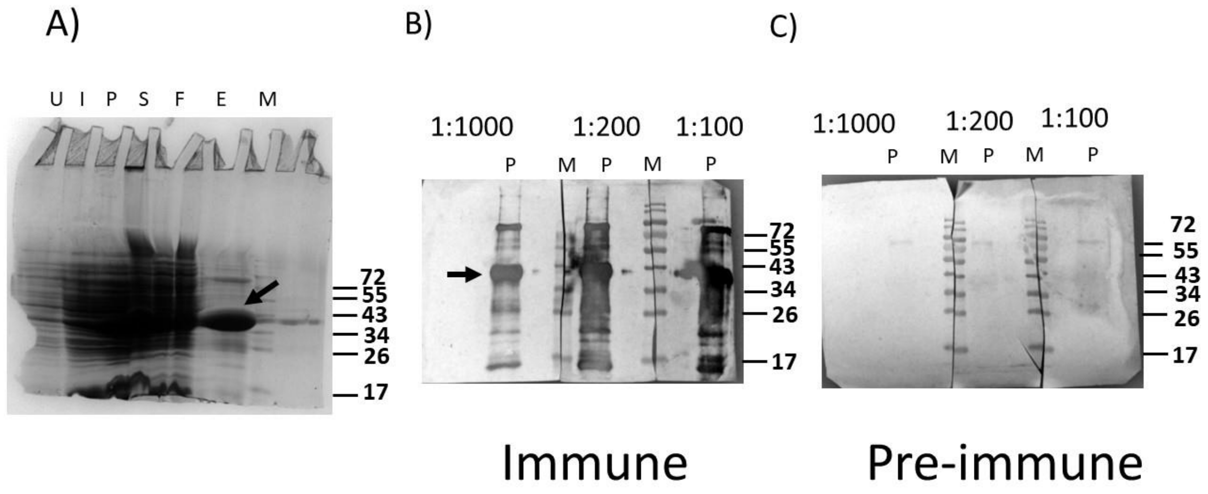

Fig S1. SDS gel electrophoresis (A) and western blot analysis using either anti-gp17 rabbit immune sera (B) or pre-immune sera (C). In (A) the lanes are as follows U (uninduced for gp17 expression), I (induced), P (pellet fraction following centrifugation of lysate), S (soluble supernatant), F (Flow through), E (eluted) and M (mol wt marker). In (B) and (C) the lanes corresponding to the purified protein are marked P. The dilutions performed are indicated above. Arrows point to the band corresponding to gp17 (34 kDa).
