# Supplementary material for: Apoptosis like symptoms associated with abortive infection of Mycobacterium smegmatis by mycobacteriophage D29
Source: PLoS One. 2022 May 17;17(5):e0259480. doi: 10.1371/journal.pone.0259480 (PMC9113562; doi:10.1371/journal.pone.0259480)
Supplement: S2 Fig — The samples analyzed are D29 infected samples along with controls (uninfected), (A and B). In (A) the changes were followed in a time dependent manner at 1 hr. intervals for 4 hrs. In (B) the same experiment was done in a MOI dependent manner. The time was interval was 2 hrs in each case. In (C) a control experiment was done using the well-known depolarizer DCCP. The exposure times are shown in minutes. Control seta are indicated as C and experimental as (E). The percent cells present in the red zones was used for the interpretation of the results (Fig 5). (PDF) [file pone.0259480.s002.pdf]

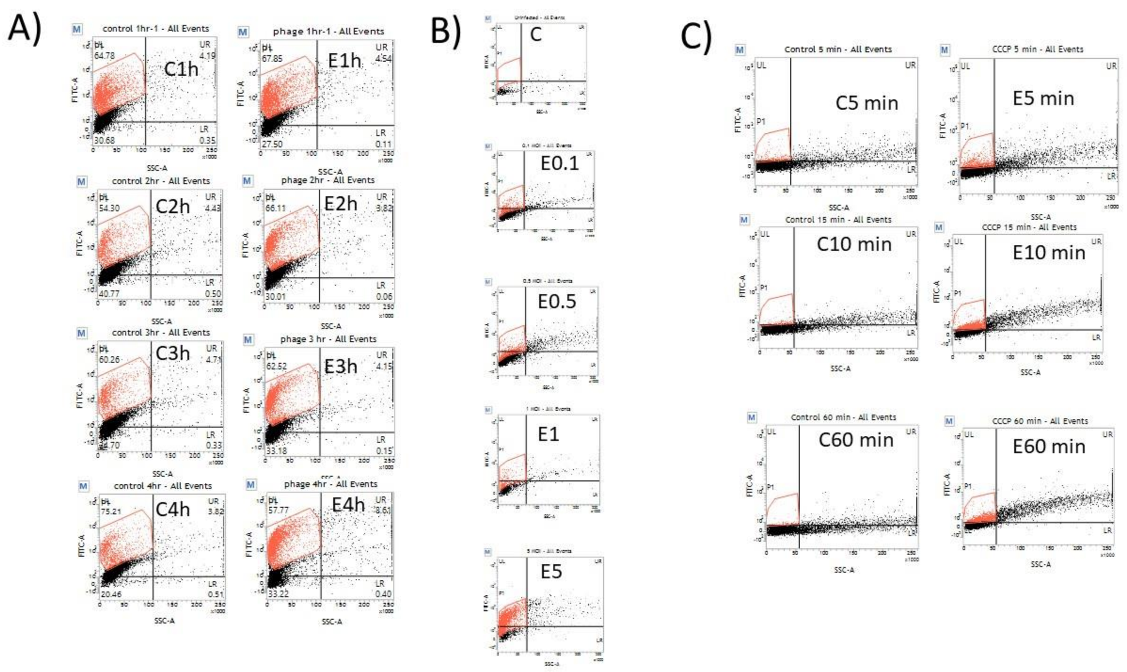

Fig S2 Raw data for FACS analysis results. The samples analyzed are D29 infected samples along with controls (uninfected), (A and B). In (A) the changes were followed in a time dependent manner at 1 hr. intervals for 4 hrs. In (B) the same experiment was done in a MOI dependent manner. The time was interval was 2 hrs in each case. In (C) a control experiment was done using the well-known depolarizer DCCP. The exposure times are shown in minutes. Control seta are indicated as C and experimental as (E). The percent cells present in the red zones was used for the interpretation of the results (Fig. 5).
