# Supplementary material for: Apoptosis like symptoms associated with abortive infection of Mycobacterium smegmatis by mycobacteriophage D29
Source: PLoS One. 2022 May 17;17(5):e0259480. doi: 10.1371/journal.pone.0259480 (PMC9113562; doi:10.1371/journal.pone.0259480)
Supplement: S3 Fig — + and–represent amplicons obtained with (+) or without reverse transcription (-). (PDF) [file pone.0259480.s003.pdf]

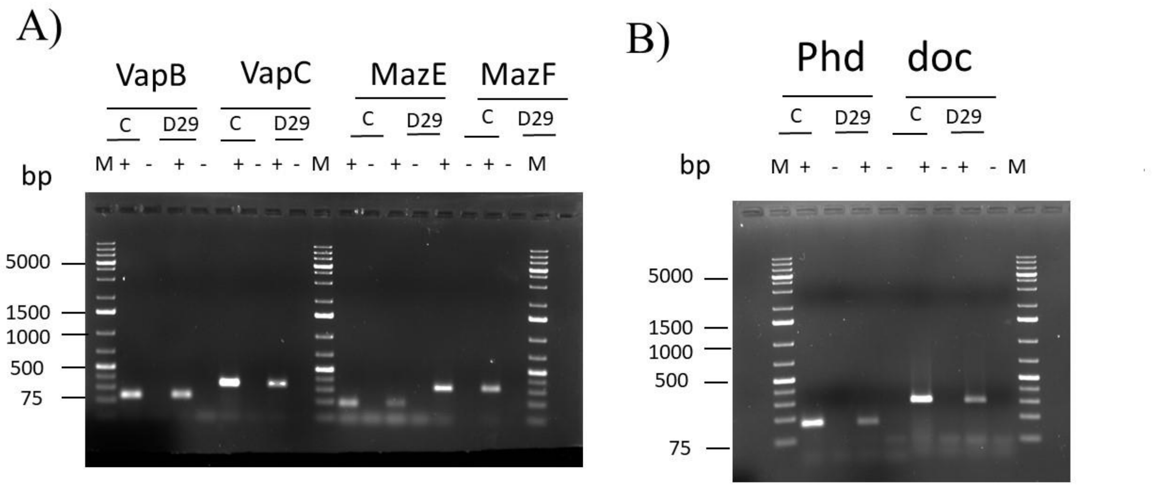

Fig S3. Agarose gel electrophoresis of RT-PCR amplicons corresponding to the indicated toxin-anti-toxin systems (Fig. 7) M represents DNA size marker. + and – represent amplicons obtained with (+) or without reverse transcription (-).
