# Supplementary material for: Apoptosis like symptoms associated with abortive infection of Mycobacterium smegmatis by mycobacteriophage D29
Source: PLoS One. 2022 May 17;17(5):e0259480. doi: 10.1371/journal.pone.0259480 (PMC9113562; doi:10.1371/journal.pone.0259480)
Supplement: S1 Table — (PDF) [file pone.0259480.s004.pdf]

Table S1. List of primers used

Target	Primer (5'-3')
Primers used for cloning the gene encoding the major head subunit gp17 of phage D29	
D2917F	CGGGATCCATGGCCGCAGGCAC
D2917R	CCCAAGCTTTCAGCCCTCGCCGC
Primers used for RT-PCR and qRT-PCR	
mazE-F	TGACCGAGTACGCCGACATC
mazE-R	GTCCCAGTCGACGGAGATCG
mazF-F	GCGCGGCGATATCTACACC
mazF-R	CGGCGATTCCCAGAAAAACC
phd-F	TCGACGAAGCCGAGATGG
phd-R	GTTGAGCTCAGCCGAACG
doc-F	GGATCGATCGCTTTTGGCGG
doc-R	AATCCAGGTCGCAGTCACGG
vapB-F	TCTAAGCATCAAACACCCGGA
vapB-R	CGTCGTAGCCCAGGATCG
vapC-F	GTTGCCATCTTGACCGACG
vapC-R	GGTGAGCCGAAAAGCCT
16S-F	CTGGGACTGAGATACGGC
16S-R	ACAACGCTCGGACCCTAC
