# Supplementary figures and images for: Apoptosis like symptoms associated with abortive infection of Mycobacterium smegmatis by mycobacteriophage D29
Source: PLoS One. 2022 May 17;17(5):e0259480. doi: 10.1371/journal.pone.0259480 (PMC9113562; doi:10.1371/journal.pone.0259480)

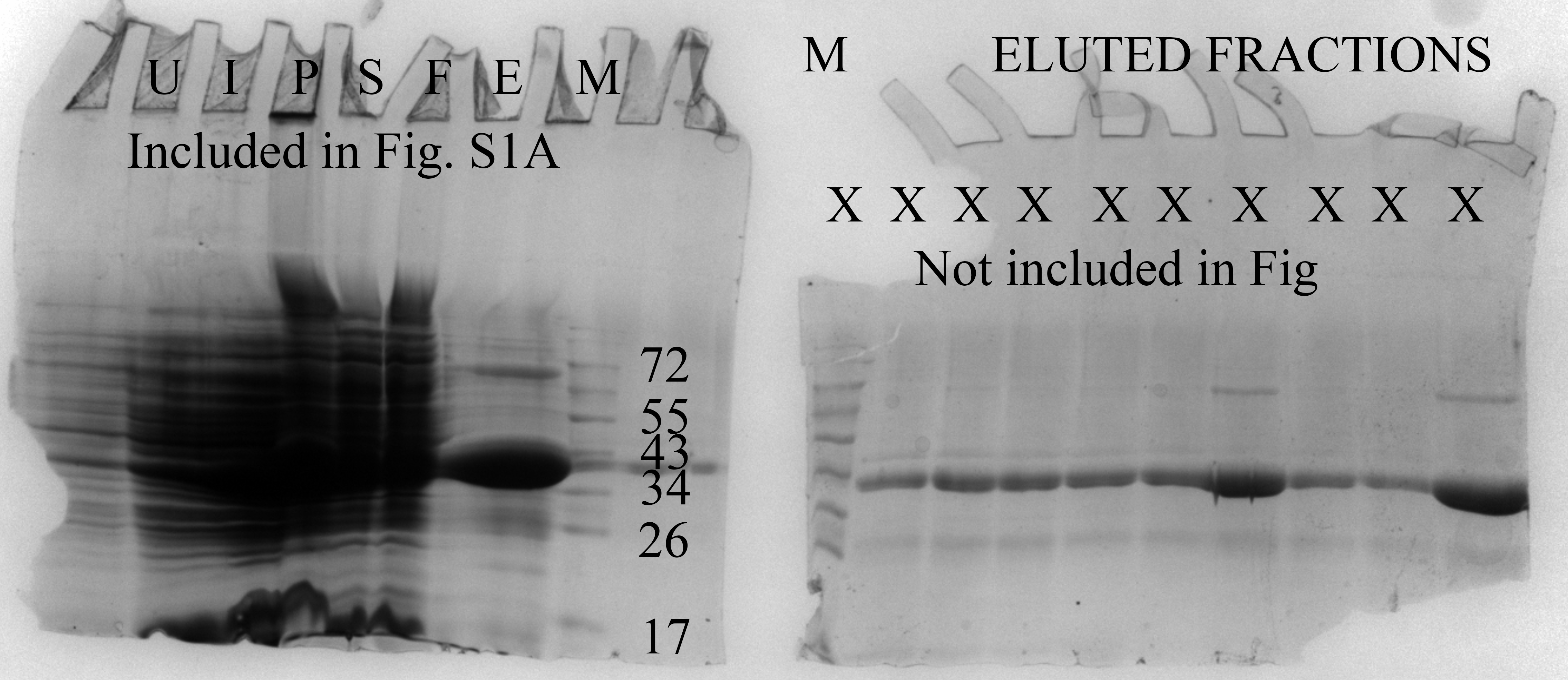

Supplement: S1 Raw image — (TIF) [file pone.0259480.s005.tif]

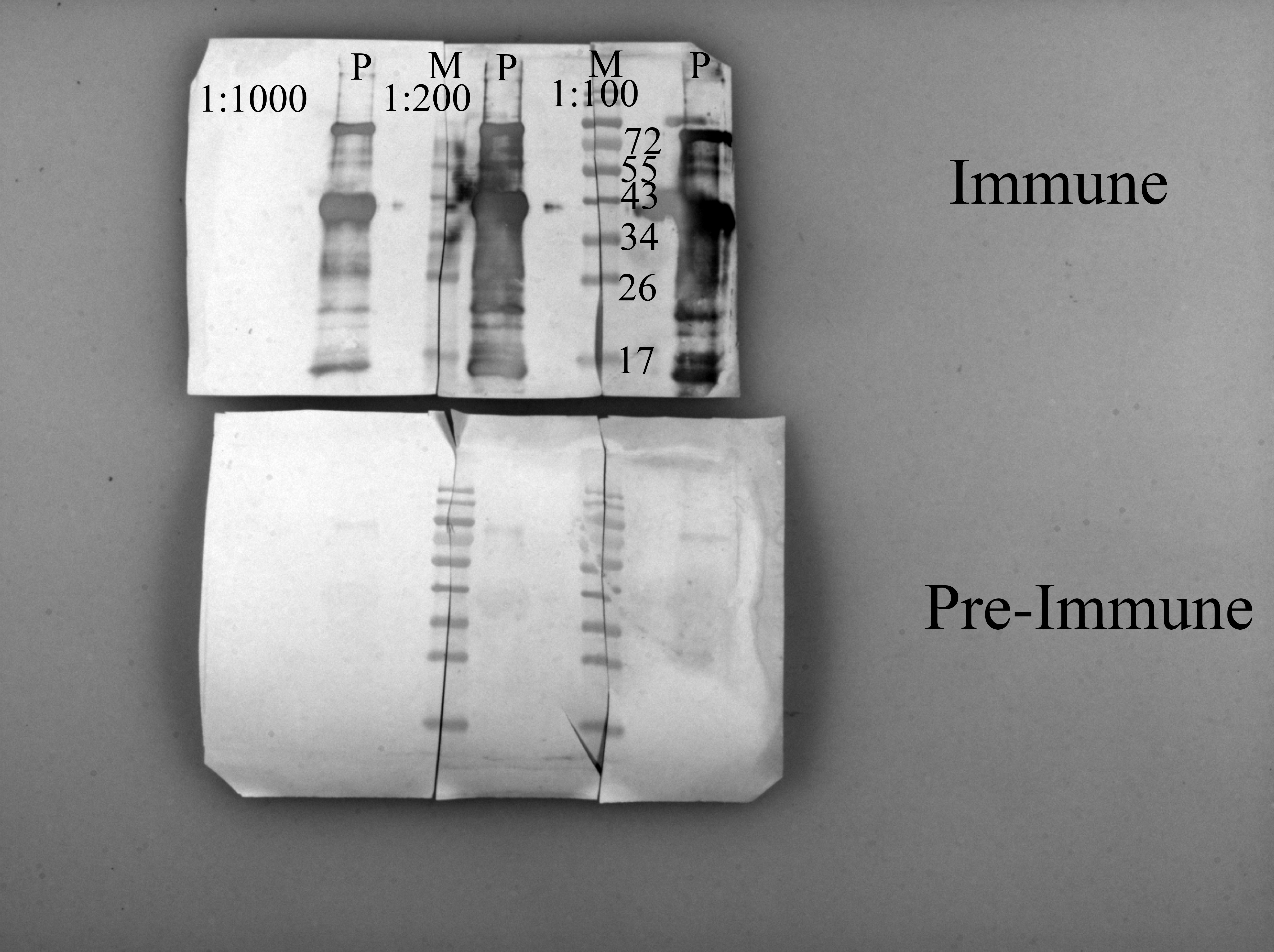

Supplement: S2 Raw image — (TIF) [file pone.0259480.s006.tif]

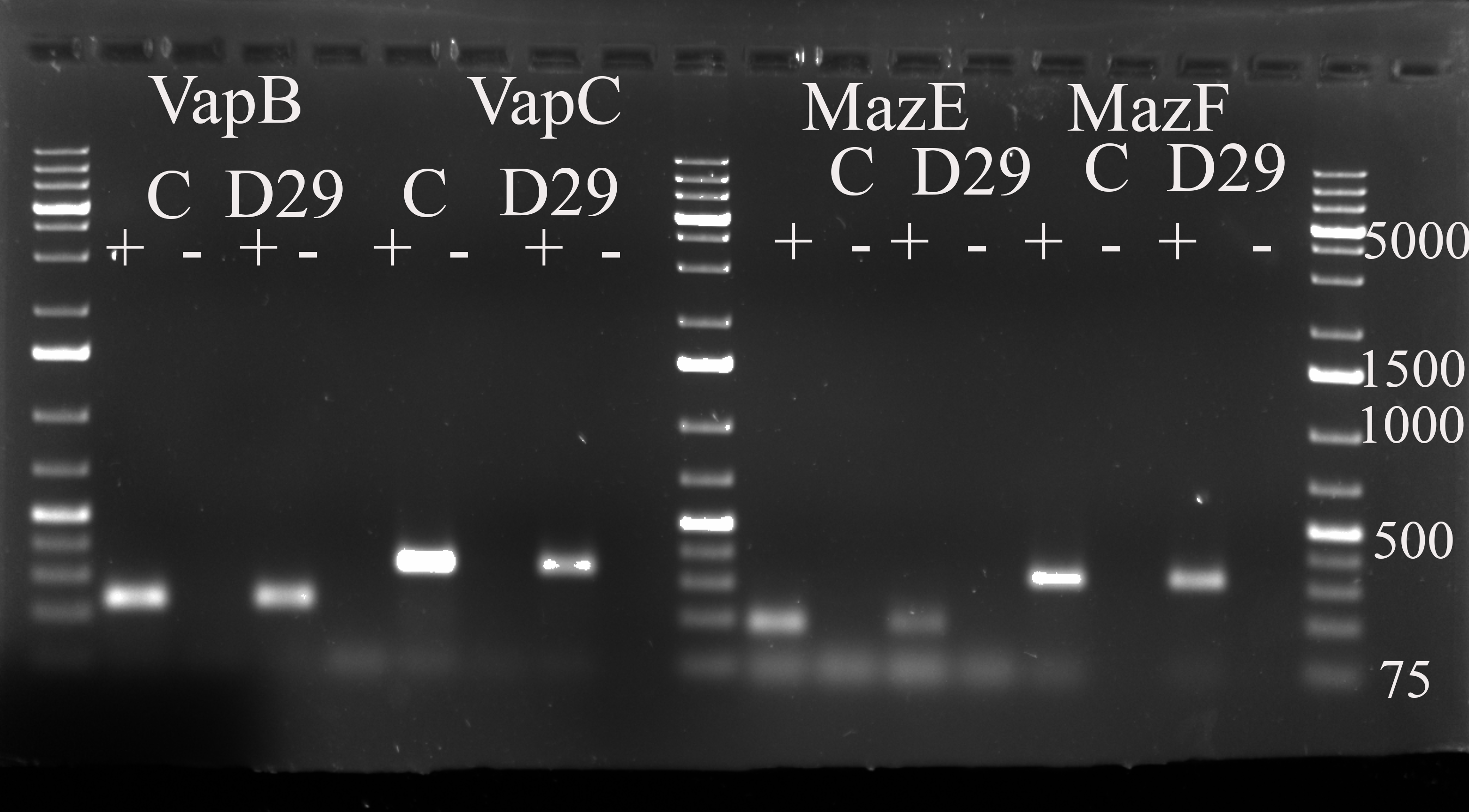

Supplement: S3 Raw image — (TIF) [file pone.0259480.s007.tif]

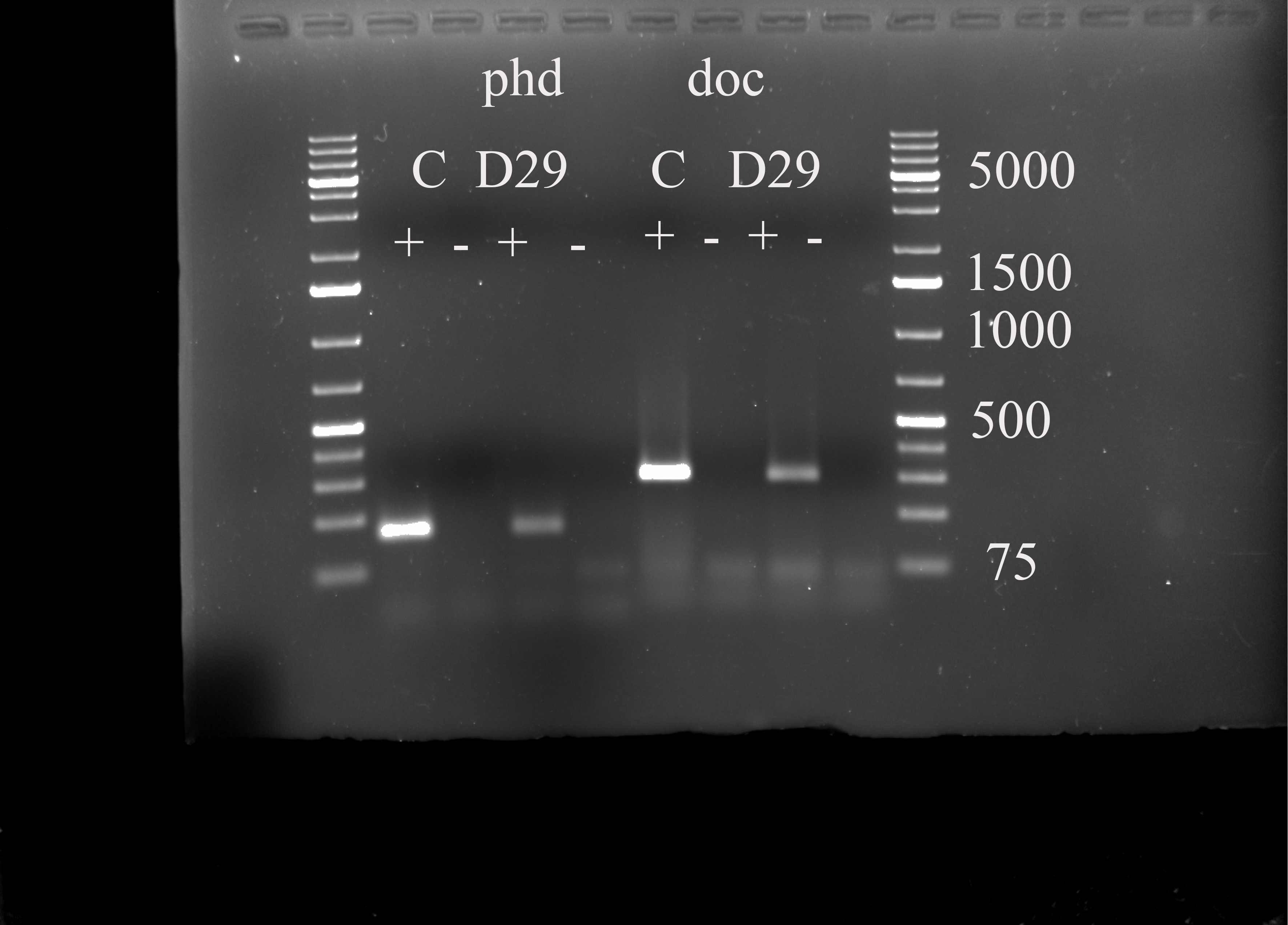

Supplement: S4 Raw image — (TIF) [file pone.0259480.s008.tif]
